# Polycystins Expression in Astrocytic Gliomas

**DOI:** 10.3390/biomedicines13040884

**Published:** 2025-04-05

**Authors:** Martha Assimakopoulou, Konstantina Soufli, Maria Melachrinou

**Affiliations:** 1 Department of Anatomy, Histology and Embryology, School of Medicine, University of Patras, 26504 Patras, Greece; kon.soufli@gmail.com; 2 Department of Pathology, School of Medicine, University of Patras, 26504 Patras, Greece; mel-par@med.upatras.gr

**Keywords:** astrocytomas, astrocytic gliomas, *PKD1*, *PKD2*, polycystin 1, polycystin 2, PC1, PC2

## Abstract

**Background:** Polycystin 1 (PC1) and polycystin 2 (PC2) proteins are members of the transient receptor potential (TRP) channels family and are encoded from *PKD1* and *PKD2* genes, respectively. Until recently, the role of *PKD1* and *PKD2* has been associated with the pathogenesis of the kidney since mutations in these genes cause autosomal dominant polycystic kidney disease (ADPKD). Recent data implicates polycystins in the pathogenesis of solid tumors. In this aspect, the expression of *PKD1* and *PKD2* in human astrocytomas is largely unknown. The aim of the present research study was to investigate the expression of *PKD1* and *PKD2* in astrocytic tumors and correlate it with clinicopathological characteristics such as the grade of malignancy, age, and gender of the patients. **Methods**: A total of 70 cases—corresponding to 8 grade II (diffuse fibrillary astrocytomas), 12 grade III (anaplastic astrocytomas), and 50 grade IV (glioblastomas multiforme)—were examined. The mRNA expression levels of *PKD1* and *PKD2* were determined through molecular qRT-PCR analysis using the relative quantification ΔΔCt method and the expression of PC1 and PC2 was detected through immunohistochemistry using the semi-quantitative H-score system. **Results**: Increased levels of *PKD1* and *PKD2* in astrocytomas were found compared with that of a normal brain (*p* < 0.05). Glioblastomas demonstrated the greatest increase in *PKD1* and *PKD2* expression compared to other grades of malignancy (*p* < 0.05). The same pattern of expression showed PC1 and PC2 proteins. A significant correlation between *PKD1* and *PKD2* as well as PC1 and PC2 expressions was found (*p* < 0.05). Although no association was detected between PC1 or PC2 and Ki67 expression (*p* > 0.05), a significant correlation between PC1 and p53 immunoexpressions, in grade III and between PC2 and p53 immunoexpressions, in grade II astrocytomas (*p* < 0.01) has emerged. PC1 expression was correlated with age of the patients (*p* < 0.05). *PKD1* and *PKD2* expression were negatively correlated with the prognosis of glioma patients. **Conclusions**: The results of this study indicate the potential involvement of polycystins in the pathogenesis of astrocytomas. However, further research is required to fully understand the mechanisms that these molecules are implicated.

## 1. Introduction

Astrocytic gliomas are the most common primary brain tumors and account for up to two-thirds of all tumors of glial origin [1]. The origin of these tumors is thought to be from neural stem or neuroglial precursor cells [2], and various mutations like mutations in the *IDH1* (isocitrate dehydrogenase) gene, co-deletion of chromosomal arms 1p/19q, and the presence or absence of the chromatin remodeling protein ATRX or mutations in the tumor suppressor gene p53 are observed [3,4]. Traditional classification of astrocytic gliomas was based on histological and immunohistochemical criteria [5]. Over the past few years, molecular changes providing a more personalized prognostic and therapeutic approach have been utilized for the classification of these tumors [4,6]. Astrocytic gliomas are divided into four grades, characterized by different prognoses. Grade I and II astrocytomas are often localized and self-limited, which indicates a possible surgical resection whereas grade III and IV astrocytomas are highly malignant and demonstrate a diffuse infiltration of the neighboring brain parenchyma and thus, an inherent tendency to recurrence and malignant progression [7,8]. Particularly, Grade IV gliomas (glioblastomas multiforme, GBMs) bear the worst prognosis with the mean survival of patients with GBM to be below 20 months despite current available therapies [8]. The low survival rate of GBM is due to the certainty of its recurrence, caused by tumor cell invasion into healthy brain tissue which makes surgical eradication unattainable and has already occurred before diagnosis [9].

The modes of glioma cell invasion include either single cell invasion which is associated with integrin-mediated adhesion of tumor cells and proteolytic extracellular matrix (ECM) degradation or collective cell invasion which is characterized by the coherent movement of groups of cells maintaining contact with each other and process potential guidance signals from the microenvironment. In collective cell invasion, the reorganization of the actin cytoskeleton in cell–cell junctional processes between tumor cells is observed [10]. Research evidence suggests that cellular structures, namely tumor microtubes (TMs), functionally connect and coordinate communication between astrocytoma cells [11]. This communication is facilitated by gap junctions, through which molecular exchange between cells including Ca^2+^ is observed. It has been shown that inhibition of intracellular Ca^2+^ waves through TMnetworks reduces the invasion capacity of glioma cells [11]. On the other hand, the tumor microenvironment (TME) of astrocytomas especially GBMs is a highly dynamic system comprising a complex multicellular component (including immune cells, endothelial cells, glial cells, and neurons) and noncellular components comprising ECM [12]. The bidirectional interactions of tumor cells with TME favoring rapid proliferation, survival, migration, and invasion of tumor cells, thus generating treatment resistance [13].

*PKD1* (*Polycystic Kidney Disease 1*) and *PKD2* (*Polycystic Kidney Disease 2*) genes that encode polycystin 1 (PC1) and polycystin 2 (PC2), respectively, are implicated in the pathogenesis of the autosomal dominant polycystic kidney disease (ADPKD) since mutations in these genes cause activation of cyst growth in kidney tubules resulting gradually in renal impairment [14]. Polycystins belong to the transient receptor potential (TRPP) subfamily of the TRP channels family. PC1 is a large (460 kDa) membrane-bound protein consisting of 11 transmembrane passes, a cytosolic C-terminal tail containing a coiled-coil domain and multiple protein cleavage sites, and an extensive extracellular N-terminal domain which acts as a mechanosensor due to its ability to recognize mechanical stimuli that allow for wide-ranging protein modifications and signaling events by converting mechanical signals into biochemical ones [15]. In addition to cell signaling, PC1 plays an important role in the assembly and stabilization of adherens junctions contributing to cell–cell interactions and interactions with the components of ECM [16,17]. Particularly, Boca and colleagues (2007) showed that PC1 may promote cell scattering and migration via actin cytoskeleton reorganization in renal epithelial cells through PI3K signaling [18]. Additionally, PC1 acts as a chemically (Ca^2+^) and mechanically sensitive protein that works in partnership with PC2 to transduce signals pressure across the cell membrane in response to flow controlling thus cellular homeostasis [19,20]. PC2 is a smaller transmembrane protein (110 kDa) also known as the TRPP2 channel. It functions as a non-selective Ca^2+^ ion channel primarily located in the endoplasmic reticulum (ER) and in multiple locations in the cell, including the plasma membrane, and the primary cilium [21]. Thus, PC2 may carry different functions depending on its subcellular localization and with which proteins it interacts [22]. Moreover, Gallagher et al. (2000) demonstrated an association between Hax-1 and the F-actin-binding protein cortactin, which suggests a link between PC2 and the actin cytoskeleton and an involvement of PC2 in the formation of cell–matrix contacts [23]. PC2, has also been implicated in regulation of the cell cycle via its calcium channel activity [24].

Previous data implicate the TRP channels in the pathogenesis of various types of tumors [25] including gliomas [26]. However, little knowledge regarding the expression of polycystins in astrocytomas exists. A single recent study by Zoi et al. (2022) provided in vitro results showing that PC1 along with hydrostatic pressure affects the expression of proteins like the mechanistic target of rapamycin (mTOR), extracellular signal-regulated kinase (ERK), focal adhesion kinase (FAK) and transcription cofactors YES-associated protein (YAP), and transcriptional coactivator with PDZ-binding motif (TAZ) as well as proteins related to anti-apoptosis, apoptosis, angiogenesis, epithelial to mesenchymal transition (EMT), and proliferation in GBM [27]. The study concludes that PC1 plays an important role in GBM mechanobiology.

The following must be considered: 1. astrocytic gliomas especially GBMs are very infiltrative tumors; 2. as mechanosensitive proteins, polycystins may be implicated in the migration of tumor cells and growth and progression of astrocytomas; and 3. previous data showing that polycystins are involved in the pathogenesis of various solid tumors [28,29,30,31,32]. The aim of the present study was to investigate the expression of *PKD1* and *PKD2* as well as PC1 and PC2 in serial sections of different grades of malignancy astrocytoma samples and compare them with normal brain tissues. The results were associated with patients’ clinicopathological characteristics.

## 2. Materials and Methods

### 2.1. Demographic Data and Neuropathology

A total of 70 Greek patients with astrocytomas (31 female, 39 males; age range, 18–82 years; mean age, 54.00 ± 15.00 years) who underwent surgery at Neurosurgery Department, School of Medicine, University of Patras over a 10-year period (2010–2020), were included in this study (Table 1). The formalin-fixed, paraffin-embedded tissue blocks were retrieved from the archives of the Department of Pathology, School of Medicine, University of Patras. The tissue material was evaluated by routine methods for histopathology, including the Ki-67 index as a proliferation marker, and graded according to the diagnostic criteria of the WHO classification system. Normal human brain tissue (cerebral hemispheres) was obtained postmortem (two males, 56 and 28 years). All ethical guidelines and rules were followed to protect patient privacy. This study has been approved by the Ethics Committee, University of Patras (6289/07-09-2020) and all processes were in accordance with the Helsinki Declaration (as revised in Edinburgh 2000).

### 2.2. Immunohistochemistry

Immunohistochemistry was performed on consecutive (semi-serial) 4 μm sections of formalin-fixed, paraffin-embedded tissue samples. Primary antibodies: rabbit polyclonal anti-polycystin 1/PC1 antibody (cat. no. ab235963; dilution 1:1300) (Abcam, Cambridge, UK), mouse monoclonal anti-polycystin-2 (clone D-3) antibody (cat. no. sc-28331; dilution 1:100) (Santa Cruz Biotechnology, Heidelberg, Germany), Ki-67 (MIB-1) (Dako, Carpinteria, CA, USA) and p53 (DO-7) (Dako, Carpinteria, CA, USA) were used. Immunohistochemical detection was carried out using the Envision Plus Detection, as previously described [32]. Positive human kidney tissue for PC1 and PC2 was included as the positive control. Additionally, PC2-immunopositive meningiomas were used as positive control for PC2 [32].

### 2.3. Scoring of Immunohistochemical Staining

To assess the fraction of immunolabeled cells in specimens from each patient case, the labeling index (LI) was determined by two observers (MA and KS), independently and blinded to sample type. This index was defined as the percentage (%) of labeled cells out of 100 tumor cells manually counted in ten non-overlapping, random fields (×400 total magnification) with the aid of an ocular grid (Table S1). Interobserver agreement for evaluation of immunostaining was within 15% (Cohen’s kappa = 0.82) [33]. Immunopositive endothelial cells were excluded from the cell counts. Microphotographs were obtained using a Nikon DXM 1200C digital camera mounted on a Nikon Eclipse 80i microscope and ACT-1C software (Nikon Instruments Inc., Melville, NY, USA, Version 1.0).

### 2.4. Gene Expression Analysis of PKD1 and PKD2 by Quantitative Real-Time PCR (qRT-PCR)

Total messenger RNA (mRNA) from normal brain and astrocytoma tissues was extracted using the commercially available kit, NucleoSpin^®^ totalRNA FFPE Kit (MACHEREY-NAGEL, GmbH & Co., Düren, Germany) as previously described [32]. For standardization of the amount of RNA, the expression of glyceraldehyde-3-phosphate dehydrogenase (GAPDH) in each sample was quantified. The sequences of each primer were obtained from published reports [27] and were synthesized by Eurofins. Sequences of primers and probes are presented in Table 2.

### 2.5. Statistical Analysis

The statistical significance of differences in expression between groups was examined with nonparametric statistical techniques using Kruskal–Wallis analysis of variance tests and the Wilcoxon rank sum post hoc tests whereas Kendall’s τ (or Spearman’s ρ) rank correlation was used to assess the significance of associations between LIs. *p* values < 0.05 were considered significant. Data analysis and graphs were prepared using a scientific data analysis and graphing platform (Prism; GraphPad Software, San Diego, CA, USA, Version 10.0).

### 2.6. Survival Analysis

Kaplan–Meier analysis was conducted with the TCGA glioblastoma and TCGA low grade glioma datasets. A cutoff value at 75% and a cutoff value at 25% to divide patients into two groups (high expression and low expression) for analyzing overall survival (OS) and progression-free survival (PFS) rates were used. The OS and RFS curve and *p* value were analyzed by log-rank test.

## 3. Results

### 3.1. Analysis of the Immunohistochemical and Gene Expression Findings

**Figure 1:**
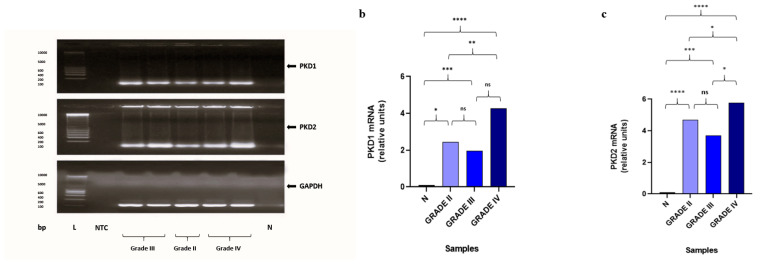
Quantitative qRT-PCR analysis of *PKD1* and *PKD2* expression using the total RNA from normal brain and astrocytoma tissues. (**a**) The product for *PKD1*, *PKD2* (149 bp) and *GAPDH* (145 bp) are indicated in agarose gel; L: DNA Ladder, N; normal brain tissue, ntc; negative template control. (**b**,**c**) The values depict the median of representative astrocytomas WHO, grade II, grade III, and grade IV from different patients. Statistical significance was calculated by Kruskal–Wallis; Statistical differences (* *p* < 0.05, ** *p* < 0.01, *** *p* < 0.001, and **** *p* < 0.0001) between different grades and normal tissues, and between different grades are indicated with asterisks, respectively. ns, non-significant.

Gene expression analysis by qRT-PCR revealed significantly increased levels of *PKD1* and *PKD2* in astrocytomas compared to normal brain tissues (*p* < 0.05). Additionally, *PKD1* expression was significantly higher in WHO, grade IV astrocytomas as compared with WHO, grade II astrocytomas (*p* < 0.05). *PKD2* expression was significantly higher in WHO, grade IV astrocytomas as compared with WHO, grade II astrocytomas and WHO, grade III astrocytomas (*p* < 0.05). Gene expression findings are presented in Figure 1. Spearman’s correlation analysis revealed a significant relationship between *PKD1* and *PKD2* expression in astrocytomas included in the study (rho = 0.629, *p* = 0.01).

**Figure 2:**
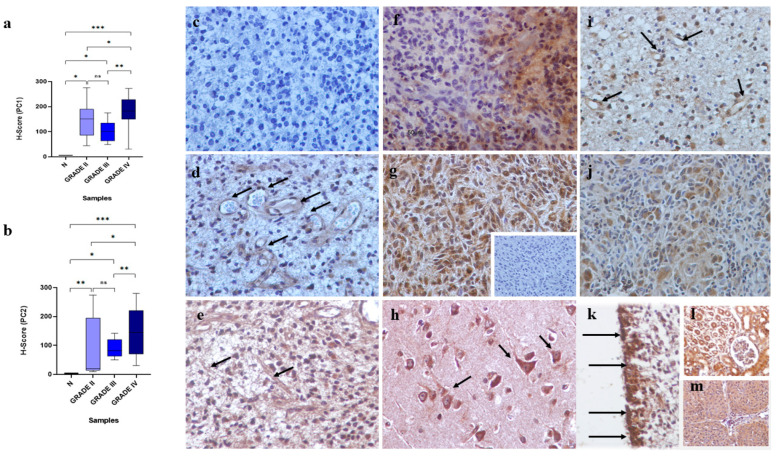
Immunoexpression of PC1 and PC2 in astrocytomas and normal brain. (**a**,**b**) A comparison of PC1 and PC2 expression between different grades of astrocytomas. The (non-parametric) Wilcoxon’s rank sum test was used and the level of significant was defined as *p* < 0.05. Statistical differences (* *p* < 0.05, ** *p* < 0.01, and *** *p* < 0.001) between different grades and normal tissues, and between different grades are indicated with asterisks, respectively. ns; non-significant. (**c**) Immunostaining is absent in negative control sections in a diffuse fibrillary astrocytoma (WHO, grade II). (**d**,**e**) Abundant cytoplasmic PC1 (LI = 275) and PC2 (LI = 116) immunoreactivity, respectively, in the same diffuse fibrillary astrocytoma (WHO, grade II). Note PC1 and PC2 immunolocalization in microvessels (*arrows*). (**f**) PC2 immunoexpression demonstrates heterogeneity in this glioblastoma multiforme (WHO, grade IV) (LI = 280). (**g**) Strong PC1 cytoplasmic immunoreactivity (LI = 229) in α glioblastoma multiforme (WHO, grade IV). Immunostaining is absent in the negative control sections in a glioblastoma multiforme (WHO, grade IV) (insert). (**h**) Representative field of the adjacent “normal” tissue in a glioblastoma multiforme (WHO, grade IV) (LI = 195 for PC1) where neurons demonstrate strong PC1 immunoreactivity. (**i**) Cytoplasmic immunostaining of PC1 in an anaplastic astrocytoma (WHO, grade III). Endothelial cells in microvessels are PC1 immunopositive (*arrows*). (**j**) Intratumoral heterogeneity for PC1 immunoexpression in a glioblastoma multiforme (WHO, grade IV); a cluster of tumor cells display PC1 immunostaining whereas most of cells are PC1 immunonegative (**k**) Expression of PC1 in α-tanycytes (*arrows*) located in the floor of the third ventricle. (**l**) Human kidney tissue was used as positive control for PC1. (**m**) Meningioma samples were used as positive control for PC2. Counterstain, hematoxylin; original magnification ×400; scale bar 50 μm.

Polycystins expression was detected in tumor cells of all astrocytomas included in this study (100%). The expression levels of PC1 were found to be higher compared to PC2 in the total of astrocytomas (*p* = 0.03). Different immunostaining profiles between different grades of malignancy were found. Specifically, WHO, grade II and III astrocytomas displayed low to moderate immunoexpression whereas most glioblastomas multiforme (WHO, grade IV) demonstrated strong immunoreactivity. Quantitative analysis of PC1 and PC2 immunoexpression between different grades of astrocytomas is presented in Table 3. A comparison of MLIs for PC1 and PC2 between different groups of patients is presented in Figure 2a,b. The PC1 and PC2 immunoreactivity was significantly greater in glioblastomas multiforme (WHO, grade IV) as compared with WHO, grade II and III astrocytomas (*p* < 0.05). In contrast, no significant difference for PC1 and PC2 immunoexpression between diffuse fibrillary astrocytomas (WHO, grade II) and anaplastic astrocytomas (WHO, grade III) was found (*p* > 0.05). Furthermore, astrocytomas displayed significantly increased immunoexpression levels for PC1 and PC2 compared with normal tissues (*p* < 0.05). Spearman’s correlation analysis revealed a significant correlation between PC1 and PC2 expressions (rho = 0.339, *p* = 0.03). The statistical significance of PC1/2 co-expression is also evident in the total of glioblastomas (rho = 0.362, *p* = 0.04). The immunolocalization of PC1 and PC2 was mainly cytoplasmic. A few cells in some samples displayed also nuclear immunolocalization. Furthermore, endothelial cells of some vessels were PC1 and/or PC2 immunopositive (Figure 2c–g).

### 3.2. Polycystins Immunoexpression in “Normal” Brain Tissue Adjacent to Tumoral Tissue and Normal Brain Samples

Neurons found in the adjacent “normal” tissue demonstrated strong PC1 and/or PC2 immunoreactivity (Figure 2h). However, normal brain specimens (cerebral hemispheres) showed low expression for polycystins in glial cells except for α-tanycytes located in the floor of the third ventricle which demonstrated strong PC1 and PC2 immunoreactivity (Figure 2i).

### 3.3. Polycystins Immunoexpression in Association with Clinicopathological Characteristics of the Patients

No association emerged between PC1 or PC2 immunoexpression and Ki67 expression (*p* > 0.05). However, there was a significant correlation between PC1 and p53 immunoexpressions, in grade III astrocytomas (*p* < 0.01) and between PC2 and p53 immunoexpressions, in grade II astrocytomas (*p* < 0.01). In grade IV astrocytomas, no significant correlation between PC1 and p53 immunoexpressions (*p* = 0.07) or PC2 and p53 immunoexpressions (*p* > 0.05) was detected. No significant correlations between PC2 immunoexpression and age of the patients (*p* > 0.05) were found. In contrast, a significant correlation between PC1 immunoexpression and the age of the patients was detected (Spearman’s rho = 0.232, *p* = 0.03) and expression levels were higher in patients older than 57 years (the median age of the patients included in the study) (*p* = 0.02). Comparison of MLIs for PC1 or PC2 between males and females demonstrated no significant difference between the groups of patients (*p* > 0.05).

### 3.4. Polycystins Expression as a Prognostic Marker in Gliomas

**Figure 3:**
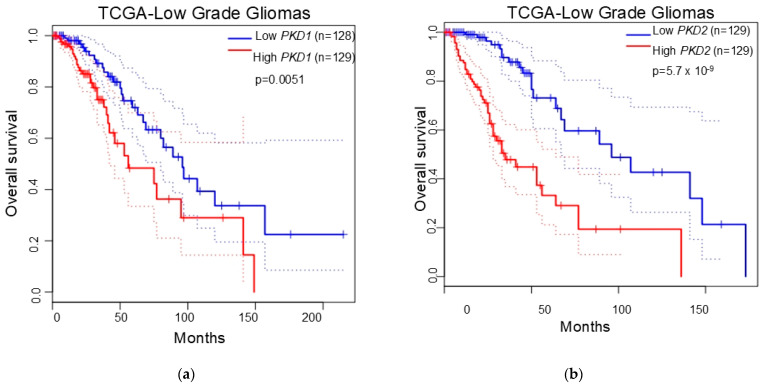
Kaplan–Meier analysis of overall survival rates of *PKD1*-low patients and *PKD1*-high patients (**a**) and *PKD2*-low patients and *PKD2*-high patients (**b**) from the TCGA low-grade gliomas dataset. All *p* values were determined using the log-rank test.

To test the possibility of polycystins as a prognostic marker for glioma patients, we analyzed the *PKD1* and *PKD2* levels in the TCGA-glioma dataset. Kaplan–Meier analysis of the overall survival (OS) rates showed that the OS rates for the low-expression group (*PKD1*-low) were significantly higher than those for the high-expression group (*PKD1*-high) (*p <* 0.001) in low-grade gliomas (Figure 3a). The OS rates for *PKD2*-low patients were significantly higher than those for *PKD2*-high patients with low-grade gliomas (*p <* 0.0001) (Figure 3b). However, the expression levels of *PKD1* and *PKD2* had no influence on the OS or PFS of glioblastoma patients.

## 4. Discussion

Polycystins are important for homeostatic maintenance in all tissues [15]. Specifically, polycystins play a dynamic role in ECM mechanosensory signaling that controls matrix production and morphogenesis. Thus, PC1 localization in focal adhesion complexes allows the protein to participate in mechanostimulatory pathways and subsequently trigger corresponding signal transduction mechanisms within the cells [16,17,18,19,20]. On the other hand, PC2 has an extensive effect on regulating Ca^2+^ levels. Ca^2+^ signaling is involved in various pathways, regulating cellular survival, which are reported to have a crucial role in cystogenesis and ADPKD development as well as in cancer [21,22,23,24,25].

In the present study, we aimed to investigate the expression of *PKD1* and *PKD2* and their encoded proteins PC1 and PC2 in astrocytoma samples including GBMs. Both PC1 and PC2 (and their counterpart genes, *PKD1* and *PKD2*) demonstrated significantly increased expression throughout the astrocytoma tissues compared to the normal brain samples. Particularly, PC1 and PC2 were expressed, mainly in the cytoplasm of tumoral cells, in all astrocytic gliomas included in the study. On the contrary, in glial cells, in normal brain tissue from cerebral hemispheres, low expression of polycystins was detected. Interestingly, in the brain samples derived from the floor of the third ventricle, a-tanycytes showed strong immunoreactivity for both polycystins confirming the presence of PC1/PC2 complex in ciliated cells [15].

Moreover, high levels of co-expression for PC1 and PC2 were found in the majority of astrocytomas. This observation highlights the possibility that polycystins may support tumor cells homeostasis in astrocytomas and probably participate in migration and invasion to neighboring structures (brain parenchyma, perivascular space, along white matter tracts, subarachnoid space) [10]. Indeed, previous data in renal cell carcinoma have revealed that PC1 becomes upregulated to promote cell migration [28]. In addition, using public basis data, *PKD1* and *PKD2* emerged as prognostic factors in low-grade gliomas since the patients with low *PKD1* or *PKD2* expression display better overall survival compared with the patients with high *PKD1* or *PKD2* expression. In colorectal cancer, high *PKD1 and PKD2* expressions have been associated with reduced recurrence-free survival and overall survival of the patients [29]. Interestingly, the comparison between six-month progression-free survival (PFS6) status good and poor (recurred within 6 months after surgery) GBM patients revealed that PKD1 is among five other genes (TRIML2, ROCK1, OBSCN, HECTD4, and ADCY7) that are significantly mutated in the poor prognosis group of GBMs providing PKD1 as a potential marker for GBM outcome [34].

**Figure 4:**
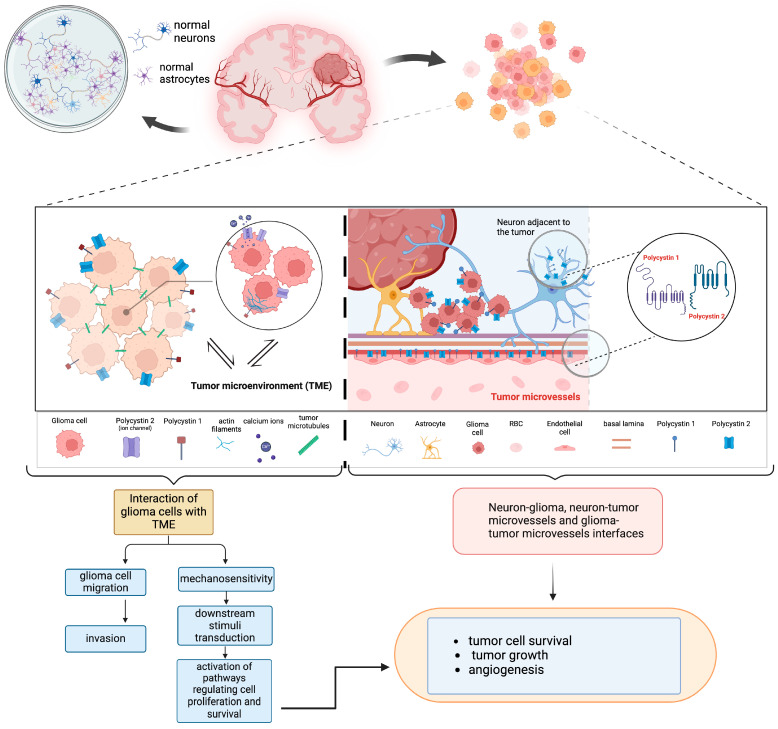
Schematic presentation of the potential role of polycystins in glioma progression.

Considering the above, the data of the present study indicates that polycystins may be implicated in oncogenic processes and the growth of astrocytic gliomas (Figure 4). Polycystins exhibited a significantly higher expression in GBMs compared to other grades of malignancy (grade II and grade III). This group of astrocytomas includes very heterogenous tumors displaying intense malignant characteristics. TME in GBM is dynamically altered by cellular composition and metabolic products as well as chemical factors, e.g., pH and oxygen levels whereas GBM cells may reprogram their TME to facilitate proliferation, and migration of tumor cells [12,13]. Previous in vitro findings report that GBM cells use PC1 as an additional mechanosensitive protein that participates in GBM development and progression [27]. Thus, polycystins may participate in the acquisition of aggressive phenotypes of GBMs through the interactions between tumor cells and TME. In addition, a significant relationship between PC1 expression and age was found. Since GBMs demonstrate a higher median age among the other astrocytomas it seems that PC1 is an additional factor that contributes to the pathogenesis of GBM. In renal cell carcinoma, PC1 and PC2 high expressions have also been associated with higher grade of malignancy [28]. Moreover, in vitro data in colorectal cancer demonstrate that both *PKD1* and *PKD2* promote an epithelial–mesenchymal transition (EMT) [29]. Furthermore, the upregulation of PC2, via activation of the Skp2/c-Myc pathway, has been associated with higher T stage and worse patient survival with nasopharyngeal carcinoma [30]. Finally, PKD1 has been shown to regulate the sensitivity of U373MG glioblastoma cells to *cis*-platinum (CDDP), a common chemotherapy agent utilized to treat gliomas [35]. Thus, the increased expression of PKD1 found in GBMs in the present study may provide a potential explanation for PKD1 contribution to the clinical chemoresistance of these tumors.

Both PC1 and PC2 were expressed in endothelial cells of mirovessels supporting the notion that polycystins may be implicated in angiogenesis in astrocytomas. It has also been shown that aberrant PC1 regulation is associated with increased angiogenesis in renal cell carcinomas [28]. Additionally, neurons in “normal” adjacent brain tissue displayed PC1 and PC2 immunoreactivity implying a role of polycystins in neuron–glioma interactions (Figure 4). Recent findings implicate neuron–glioma interfaces in tumor growth [13]. Specifically, Venkatarami and colleagues (2019, 2022) have reported functionally active neural-glioma synapses which exhibit Ca^2+^ activity in response to neural activity and favor the promotion of tumor cell invasiveness. Interestingly, migration of tumor cells is dependent on the frequency of Ca^2+^ signals [36,37]. Although the findings of the present study suggest the potential involvement of polycystins in the pathogenesis of astrocytomas, confirmation in a larger number of patients is needed.

## 5. Conclusions

The association of polycystins with the invading tumor cells may represent a novel therapeutic target for gliomas. Further studies will clarify the key elements of multifaceted mechanisms in the development and growth of these tumors that involve polycystins.

## Figures and Tables

**Table 1:** Clinical information of study cohort.

Clinical Characteristics	Histological Classification (WHO)		
	Diffuse Fibrillary Astrocytomas (WHO, Grade II)	Anaplastic Astrocytomas (WHO, Grade III)	Glioblastomas Multiforme (WHO, Grade IV)
*No. of patients*	8	12	50
*Age (years)*			
Mean ± SD (range)	47.25 ± 12.61 (27–64)	49.08 ± 18.32 (18–75)	57.29 ± 13.47 (30–82)
≥60	1 (12.50%)	5 (41.66%)	22 (44.00%)
*Sex*			
Male	5 (62.50%)	8 (66.67%)	26 (52%)
Female	3 (37.5%)	4 (33.33%)	24 (48%)
*Tumor location*			
supratentorial	8 (100%)	12 (100%)	50 (100%)
not supratentorial	0 (0%)	0 (0%)	0 (0%)
*Prior treatment*	0 (0%)	0 (%)	2 (4%)
*Primary/recurrent tumors*	1 (12.5%)/7 (87.5%)	1 (8.33%)/11 (91.67%)	10 (20%)/40 (80%)

**Table 2:** Sequences of primers.

Gene	Primer	Sequence 5′-3′
*PKD1*	Forward Reverse	CAAGACACCCACATGGAAACG CGCCAGCGTCTCTGTCTTCT
*PKD2*	Forward Reverse	GCGAGGTCTCTGGGGAAC TACACATGGAGCTCATCATGC
*GAPDH*	Forward Reverse	GAGTCAAGCGATTTGGTCGT TTGATTTTGGAGGGATCTCG

**Table 3:** Quantitative analyses of immunohistochemical expression for PC1 and PC2 using the labeling index (LI) in astrocytic gliomas *.

Tumor Group	Diffuse Fibrillary Astrocytomas (WHO, Grade II)		Anaplastic Astrocytomas (WHO, Grade III)		Glioblastomas Multiforme (WHO, Grade IV)	
	PC1	PC2	PC1	PC2	PC1	PC2
**MLI, %**	150.00 ^a^	20.00	101.00	82.00	182.00 ^b^	130.00
**IQR, %**	85.00–190.00	13.50–195.00	63.00–135.00	62.50–120.50	149.25–228.25	50.50–220.50
**Minimum** **LI, %**	44.00	10.00	48.00	50.00	31.00	10.00
**Maximum** **LI, %**	275.00	274.00	175.00	141.00	272.00	280.00

* No., the number of tumor specimens, each corresponding to a tumor case; MLI, median labeling index; IQR, interquartile range which is delimited by the 25th and 75th population percentiles; LI, labeling index; and WHO, World Health Organization. The letter a indicates PC1 expression vs. PC2 expression in diffuse fibrillary astrocytomas (WHO, grade II), *p* = 0.04. The letter b indicates PC1 expression vs. PC2 expression in glioblastomas multiforme (WHO, grade IV), *p* = 0.04.

## Data Availability

The original contributions presented in this study are included in the article/Supplementary Material. Further inquiries can be directed to the corresponding author.

## Supplementary Materials

The following supporting information can be downloaded at: https://www.mdpi.com/article/10.3390/biomedicines13040884/s1, Table S1: Scoring of immunohistochemical staining for PC1, PC2, Ki67, and p53 defined as Labeling Index (LI). LI represents the percentage (%) of labeled cells out of 100 tumor cells manually counted in ten non-overlapping, random fields (×400 total magnification), in each sample. ND: Not detected due to lack of serial sections.
