# Supplementary material for: Polycystins Expression in Astrocytic Gliomas
Source: Biomedicines. 2025 Apr 5;13(4):884. doi: 10.3390/biomedicines13040884 (PMC12025129; doi:10.3390/biomedicines13040884)
Supplement: Supplementary file 1 [file biomedicines-13-00884-s001.zip › biomedicines-3520724-supplementary.pdf]

Supplementary Material

Table S1. Scoring of immunohistochemical staining for PC1, PC2, Ki67, and p53 defined as Labeling Index (LI). LI represents the percentage (%) of labeled cells out of 100 tumor cells manually counted in ten non-overlapping, random fields (X400 total magnification), in each sample. ND: Not detected due to lack of serial sections

Grade II

PC1	PC2	Ki67	p53
44,00	ND	ND	ND
174,00	10,00	ND	0
190,00	274,00	2	70
150,00	20,00	4	10
85,00	ND	1	ND
275,00	116,00	2	ND
128,00	17,00	5	2
150,00	20,00	10	30

Grade III

135,00	ND	ND	ND
48,00	100,00	15	25
115,00	ND	ND	ND
63,00	82,00	ND	50
175,00	141,00	40	120
101,00	50,00	10	70
100,00	75,00	ND	80
63,00	ND	ND	10
101,00	ND	ND	ND

135,00	ND	ND	ND
135,00	ND	ND	ND
60,00	ND	ND	5
Grade IV			
230,00	ND	ND	ND
150,00	220,00	ND	ND
155,00	233,00	ND	ND
149,00	270,00	ND	ND
216,00	ND	ND	ND
125,00	ND	ND	ND
240,00	ND	20	ND
270,00	ND	ND	ND
226,00	ND	10	ND
77,00	221,00	25	ND
229,00	ND	30	ND
55,00	ND	ND	ND
272,00	55,00	60	90
270,00	40,00	ND	ND
225,00	280,00	10	ND
193,00	40,00	ND	ND
213,00	ND	ND	ND
105,00	ND	ND	ND
ND	145,00	20	10
210,00	90,00	ND	ND

115,00	180,00	ND	ND
31,00	150,00	30	ND
100,00	118,00	ND	ND
180,00	280,00	ND	ND
171,00	113,00	ND	ND
246,00	ND	ND	ND
145,00	30,00	40	ND
54,00	121,00	ND	ND
109,00	46,00	ND	ND
205,00	ND	ND	ND
244,00	ND	ND	ND
ND	188,00	20	ND
ND	40,00	30	ND
170,00	70,00	30	ND
158,00	263,00	30	ND
151,00	245,00	30	ND
230,00	130,00	ND	ND
190,00	80,00	ND	ND
195,00	216,00	ND	ND
244,00	71,00	ND	ND
174,00	200,00	20	0
210,00	160,00	30	80
175,00	ND	30	1
235,00	ND	35	70

156,00	ND	25	10
184,00	ND	20	0
152,00	30,00	ND	2
152,00	10,00	10	ND
182,00	42,00	30	ND
240,00	244,00	20	80
